# HYDROchlorothiazide versus placebo to PROTECT polycystic kidney disease patients and improve their quality of life: study protocol and rationale for the HYDRO-PROTECT randomized controlled trial

**DOI:** 10.1186/s13063-024-07952-x

**Published:** 2024-02-14

**Authors:** Thomas Bais, Esther Meijer, Bart J. Kramers, Priya Vart, Marc Vervloet, Mahdi Salih, Bert Bammens, Nathalie Demoulin, Polina Todorova, Roman-Ulrich Müller, Jan Halbritter, Alexander Paliege, Emilie Cornec-Le Gall, Bertrand Knebelmann, Roser Torra, Albert C. M. Ong, Fiona E. Karet Frankl, Ron T. Gansevoort

**Affiliations:** 1 Department of Nephrology, University Medical Center Groningen, Groningen, The Netherlands; 2 Department of Clinical Pharmacy and Pharmacology, University Medical Center Groningen, Groningen, The Netherlands; 3 Department of Nephrology, Amsterdam University Medical Centers, Location Vrije Universiteit Amsterdam, Amsterdam, The Netherlands; 4 Department of Internal Medicine, Division of Nephrology and Transplantation, Erasmus University Medical Center, Rotterdam, The Netherlands; 5 Department of Nephrology, Dialysis and Renal Transplantation, University Hospitals Leuven, Leuven, Belgium; 6 Division of Nephrology, Cliniques Universitaires Saint-Luc, Brussels, Belgium; 7 University of Cologne, Faculty of Medicine and University Hospital Cologne, Department 2 for Internal Medicine, Cologne, Germany; 8 Department of Nephrology, Charité Universitätsmedizin Berlin, Berlin, Germany; 9 Department of Nephrology, Universitätsklinikum Carl Gustav Carus Dresden, Dresden, Germany; 10 University Brest, Inserm, UMR 1078, GGB, Brest, 29609 France; 11 Service de Néphrologie, Hémodialyse et Transplantation Rénale, CHRU Brest, Brest, 29609 France; 12 Department of Nephrology, Necker–Enfants Malades Hospital AP-HP, Paris, France; 13 Inherited Kidney Diseases, Nephrology Department, Fundació Puigvert, Institut d’Investigació Biomèdica Sant Pau (IIB-SANT PAU), Barcelona, Spain; 14 Academic Nephrology Unit, Department of Infection, Immunity and Cardiovascular Disease, University of Sheffield, Sheffield, UK; 15 Department of Medical Genetics and Division of Renal Medicine, University of Cambridge, Cambridge, UK; 16 Division of Nephrology, Department of Internal Medicine, University Medical Center Groningen, PO Box 30.001, 9700 RB Groningen, The Netherlands

## Abstract

**Background:**

Autosomal dominant polycystic kidney disease (ADPKD) leads to progressive renal cyst formation and loss of kidney function in most patients. Vasopressin 2 receptor antagonists (V2RA) like tolvaptan are currently the only available renoprotective agents for rapidly progressive ADPKD. However, aquaretic side effects substantially limit their tolerability and therapeutic potential. In a preliminary clinical study, the addition of hydrochlorothiazide (HCT) to tolvaptan decreased 24-h urinary volume and appeared to increase renoprotective efficacy. The HYDRO-PROTECT study will investigate the long-term effect of co-treatment with HCT on tolvaptan efficacy (rate of kidney function decline) and tolerability (aquaresis and quality of life) in patients with ADPKD.

**Methods:**

The HYDRO-PROTECT study is an investigator-initiated, multicenter, double-blind, placebo-controlled, randomized clinical trial. The study is powered to enroll 300 rapidly progressive patients with ADPKD aged ≥ 18 years, with an eGFR of > 25 mL/min/1.73 m^2^, and on stable treatment with the highest tolerated dose of tolvaptan in routine clinical care. Patients will be randomly assigned (1:1) to daily oral HCT 25 mg or matching placebo treatment for 156 weeks, in addition to standard care.

**Outcomes:**

The primary study outcome is the rate of kidney function decline (expressed as eGFR slope, in mL/min/1.73 m^2^ per year) in HCT versus placebo-treated patients, calculated by linear mixed model analysis using all available creatinine values from week 12 until the end of treatment. Secondary outcomes include changes in quality-of-life questionnaire scores (TIPS, ADPKD-UIS, EQ-5D-5L, SF-12) and changes in 24-h urine volume.

**Conclusion:**

The HYDRO-PROTECT study will demonstrate whether co-treatment with HCT can improve the renoprotective efficacy and tolerability of tolvaptan in patients with ADPKD.

**Supplementary Information:**

The online version contains supplementary material available at 10.1186/s13063-024-07952-x.

## Introduction

**Fig. 1:**
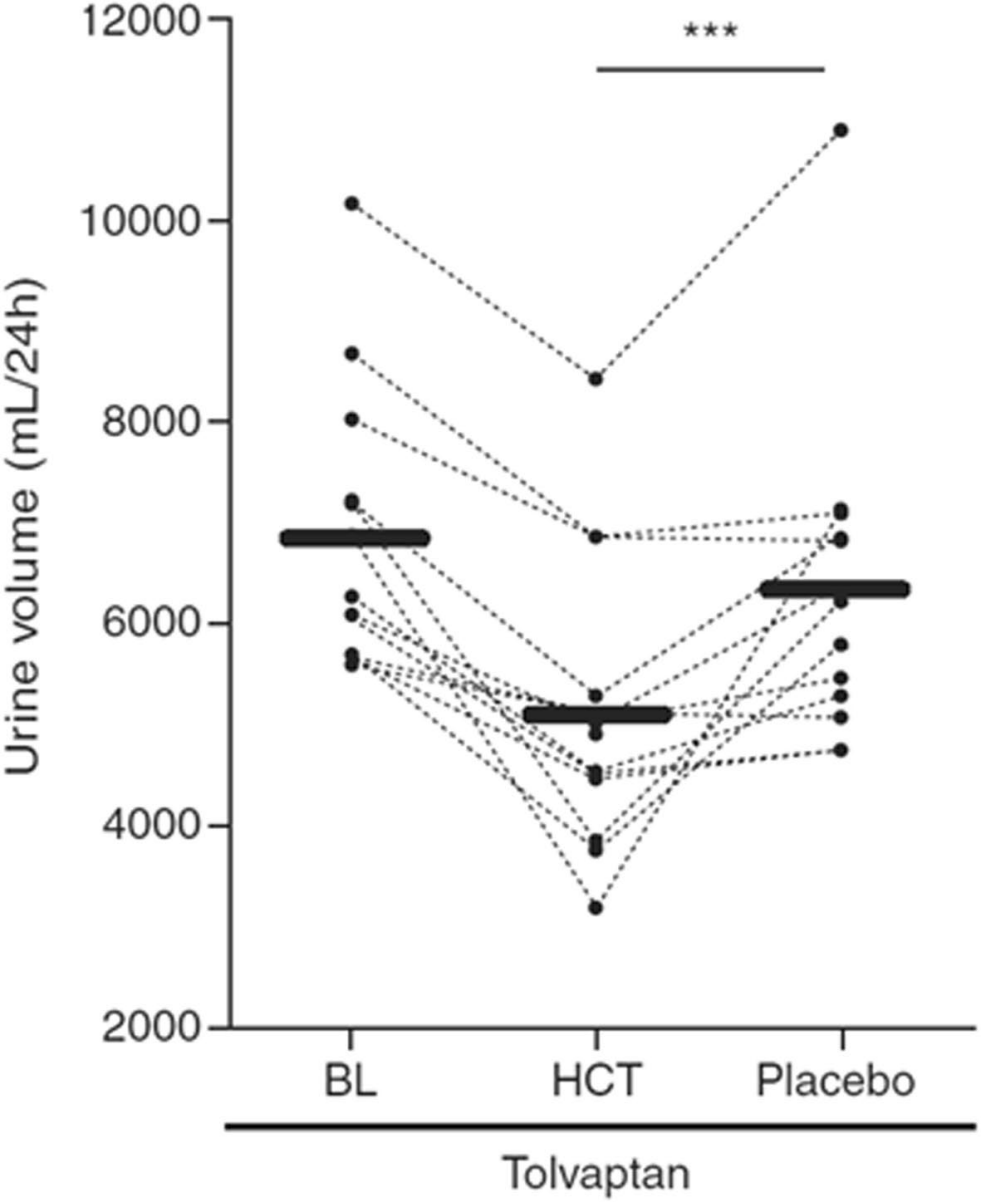
Administration of HCT in tolvaptan-treated ADPKD patients significantly reduces 24-h urine volume compared to baseline and placebo. HCT and placebo treatment were given in random order. BL, baseline visit. ***P < 0.01. Adapted from Kramers et al. [17]

Autosomal dominant polycystic kidney disease (ADPKD) is the most common hereditary kidney disease and leads to progressive renal cyst formation, to loss of renal function, and frequently to kidney failure [1–3]. In the majority of patients, ADPKD is caused by a mutation in the *PKD1* or *PKD2* gene, which leads to the aberrant functioning of the polycystin complex that is mainly located on the primary cilium of renal tubular cells. This stimulates cyclic AMP (cAMP) production in these cells and promotes cystogenesis [4]. In animal studies, vasopressin type 2 receptor antagonists (V2RAs) exert beneficial effects on ADPKD disease progression by reducing cAMP production and decreasing proliferative pathway signaling in these cells [5, 6]. In ADPKD patients at high risk of rapid disease progression, it has been demonstrated that treatment with the V2RA tolvaptan slows the rate of renal function decline in relatively early-stage disease [7] (TEMPO 3:4 trial, phase 3), later-stage disease [8] (REPRISE trial, phase 3), and real-world phase 4 study [9]. These renoprotective properties led to marketing authorization in Europe and the USA for tolvaptan in ADPKD with a high likelihood of rapid disease progression, making it the first and as yet only renoprotective treatment for these patients. Despite its proven efficacy, the use of tolvaptan is significantly hampered by its aquaretic side effects, principally polyuria, nocturia, and thirst. In the TEMPO 3:4 trial, 78% of patients receiving tolvaptan reported at least one aquaretic side effect [10]. Of the tolvaptan-treated group, 23% did not complete the 3-year trial [7]. In addition, 45% of tolvaptan-treated patients who did complete the TEMPO 3:4 trial could not tolerate the target dose of 120 mg daily, largely due to aquaretic side effects. Furthermore, a post hoc analysis of that trial showed that young patients with better-preserved kidney function were more likely to discontinue tolvaptan treatment [10]. Another study demonstrated that 24-h urine volume was positively correlated with baseline eGFR in ADPKD patients treated with tolvaptan [11]. Taken together, these findings suggest that young patients with relatively high eGFR are most likely to discontinue tolvaptan treatment due to aquaretic side effects. This is unfortunate, because these patients are predicted to benefit the most from long-term treatment [12]. Considering that tolvaptan is presently the only available renoprotective treatment for ADPKD, it is important to develop strategies to improve its tolerability. A promising approach is to co-prescribe hydrochlorothiazide (HCT). Tolvaptan treatment impairs the vasopressin 2 receptor and thereby decreases renal water reabsorption. The function of this receptor is also impaired in the various forms of nephrogenic diabetes insipidus (NDI) [13]. HCT has been a cornerstone of pharmacological treatment for NDI for decades [14–16]. It therefore seems rational to use HCT to limit aquaresis in tolvaptan recipients, who in essence have an iatrogenic form of NDI. In our phase 1, randomized, placebo-controlled trial, the hypothesis was tested whether HCT could also reduce aquaresis in tolvaptan recipients. In this study of 13 tolvaptan-treated ADPKD patients, co-treatment with HCT was safe, reduced 24-h urine volume by 26% (Fig. 1), and improved quality of life (QoL) [17]. Furthermore, treatment with HCT reduced plasma copeptin levels (a surrogate marker of vasopressin production) and reduced the urinary excretion of monocyte chemoattractant protein-1 (MCP-1), an inflammatory biomarker that is associated with accelerated ADPKD disease progression [17, 18]. In addition, an animal model demonstrated a trend towards lower cystic index in tolvaptan/HCT-treated mice compared to tolvaptan monotherapy (*p* = 0.06) [17]. Thus, along with improving urine production and quality of life during tolvaptan treatment, these findings suggest an additive renoprotective effect of co-treatment with HCT. However, the short-term nature of these studies precludes any conclusions on longer-term effects. We have designed the HYDRO-PROTECT study (HYDROchlorothiazide to PROTECT polycystic kidney disease patients and improve their quality of life) to evaluate the effect of longer-term co-treatment with HCT on renoprotective efficacy and tolerability of tolvaptan in patients with rapidly progressive ADPKD.

## Methods

### Study setting and population

The HYDRO-PROTECT study is an investigator-driven, randomized, double-blind, placebo-controlled, clinical trial in 300 participants with ADPKD receiving tolvaptan. The trial will include individuals with an ADPKD diagnosis based on the modified Pei-Ravine criteria [19], aged ≥ 18 years, with an eGFR > 25 mL/min/1.73 m^2^, and on stable treatment with the highest tolerated dose of tolvaptan for a minimum of 3 months. Since only patients who are being treated with tolvaptan are recruited, who by indication should have a high risk for rapid disease progression, no additional inclusion criteria for rapidly progressive disease are applied. Detailed inclusion and exclusion criteria are listed in Table 1. The trial is designed as a multicenter international study in 13 sites across Europe (located in the Netherlands, Belgium, Germany, France, and Spain) and the UK. Patients will be selected from the outpatient nephrology departments of the participating centers. This study has been presented at ADPKD patient gatherings and was featured in a Dutch patient journal for kidney disease to help reach the target sample size.

**Table 1:** Eligibility criteria for the HYDRO-PROTECT study

**Inclusion** - ADPKD diagnosis (based on the modified Pei-Ravine criteria) - ≥ 18 years old - eGFR > 25 mL/min/1.73 m^2^ (in Belgium: eGFR > 35 mL/min/1.73 m^2^) - On stable treatment with the highest tolerated dose of tolvaptan for a minimum of 3 months **Exclusion** - Known intolerance to hydrochlorothiazide - Diabetes mellitus type 1 or type 2 - Hypokalemia (< 3.5 mmol/L) - Current use of any diuretic - Changes in antihypertensive treatment for one month prior to inclusion - Orthostatic hypotension or BP < 105/65 mmHg - Uncontrolled hypertension (BP > 160/100 mmHg) - History of active gout (defined as ≥ 2 episodes during the last year) despite preventive treatment for gout (allopurinol, desuric, febuxostat, and/or colchicine) - History of skin cancer (basal cell carcinoma, squamous cell carcinoma, or melanoma) - Primary renal disease other than ADPKD - Pregnancy	

### Study design

After obtaining informed consent and completing the screening visit, patients who meet the eligibility criteria will be included in the study. When baseline measurements have been performed, patients will be randomly assigned to daily oral HCT 25 mg or matching placebo, in addition to standard care. To minimize imbalance between treatment groups, stratified randomization (1:1) by minimization (Pocock and Simon method) will be performed using an online randomization tool provided by the UMC Groningen, with stratification for eGFR (< and ≥ 45 mL/min/1.73 m^2^), age (< and ≥ 45 years), and participating center. Minimization is a dynamic randomization method that minimizes imbalance over selected prognostic factors [20]. The planned recruitment period is approximately 2 years.

**Fig. 2:**
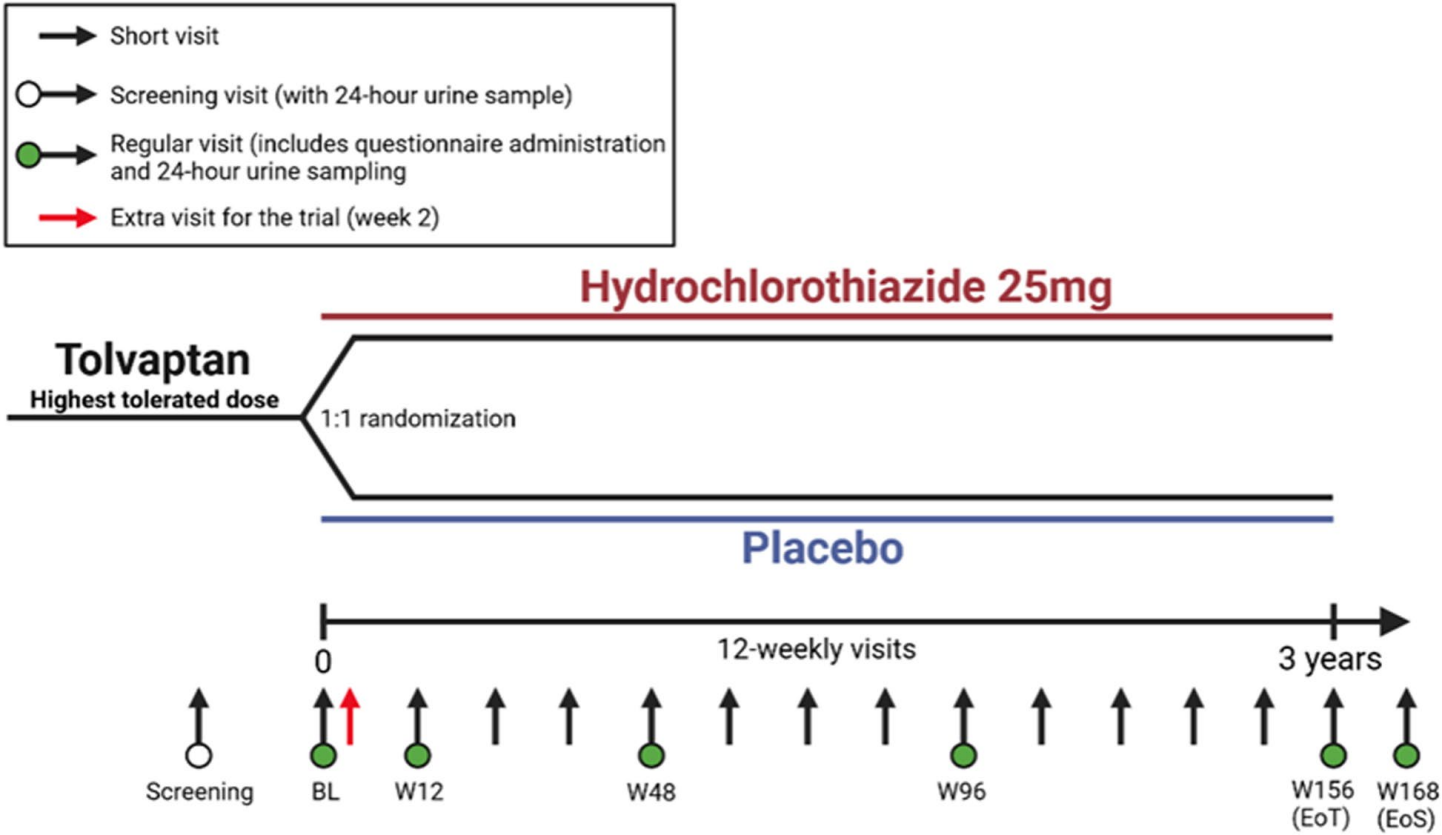
Schematic presentation of the HYDRO-PROTECT visit schedule. SV, screening visit; BV, baseline visit; EoT, end of treatment; EoS, end of study

Patients with ADPKD who are treated with tolvaptan are monitored regularly because of potential hepatotoxic reactions to tolvaptan. The HYDRO-PROTECT study is designed to be pragmatic; where possible, study measurements will be performed during routine 12-weekly clinical visits to minimize patient burden. Figure 2 presents a schematic overview of the study visit schedule. Patients will be randomized during the baseline visit (week 0 (W0)), and study treatment is started directly after this visit. Two weeks after the start of treatment (W2), an additional study visit (or a local laboratory visit combined with a telephone call) will be performed to monitor side effects, blood pressure, and potential serum electrolyte changes. All other study measurements will be conducted during the routine 12-weekly visits. Preferably, these visits should be performed at the participating medical center, although some may be performed locally for pragmatic reasons. During these study visits, blood samples are drawn and blood pressure will be measured after drug intake. Additionally, 24-h urine samples will be collected, and questionnaires will be completed during the regular study visits. An overview of the different study measurements at various time points is presented in Table 2. Study treatment ends at week 156 with the end-of-treatment (EoT) visit, and study participation ends at week 168 after the end-of-study (EoS) visit. We expect that the patient-centric approach of incorporating study measurements in routine clinical visits and allowing the W2 safety visit to be performed at a local laboratory will maximize patient adherence.

**Table 2:** Overview of the study measurements of the HYDRO-PROTECT trial

Visit	SV	BV	Additional safety assessment	Regular visit	Short visit	EoT	EoS/EET
**Week**	− 8 to − 2	0	2 (± 2 days)	12, 48, 96, 156 (± 2 weeks)	24, 36, 60, 72, 84, 108, 120, 132, 144 (± 2 weeks)	156 (± 2 weeks)	168 (± 2 weeks)
**Activity**							
Eligibility and informed consent	X						
Randomization		X					
Vital signs	X	X		X	X*	X	X
Physical examination		X		X**	X**	X	X
Laboratory assessment 1***			X				
Laboratory assessment 2***	X				X		
Laboratory assessment 3***		X		X		X	X
Biobanking		X		Week 12			
24-h urine collection	X	X		X		X	X
Questionnaires		X		X		X	X
Drug accountability				X	X	X	
Safety assessment		X	X	X	X	X	X

*SV* screening visit, *BV* baseline visit, *EoT* end of treatment, *EoS* end of study, *EET* early-end of treatment

*Vital signs for short visits can be measured by the patient at home

**Physical examination during short and regular study visits will only be performed when deemed clinically necessary by the attending physician

***Laboratory assessment 1: serum sodium, potassium, creatinine, and urea. Laboratory assessment 2: laboratory assessment 1 plus serum hemoglobin, ASAT, ALAT, bilirubin, magnesium, and uric acid. Laboratory assessment 3: laboratory assessment 2 plus serum total calcium, phosphate, albumin, HbA1c, cholesterol (total, LDL, and HDL), glucose, venous blood gas, and 24-h urine volume and sodium, creatinine, albumin, urea, and potassium concentrations

### Study measurements and data collection

Study measurements consist of blood analysis, measurement of vital parameters, and for some visits also 24-h urine sample collection and completion of QoL-related questionnaires. An overview can be found in Table 2.

#### Biochemical analyses

Blood analysis will be performed at every study visit. Most blood measurements are part of standard care for all tolvaptan-treated ADPKD patients, including 12-weekly eGFR, serum electrolytes, and liver function tests. For safety purposes, an additional laboratory assessment will be performed 2 weeks after the start of study treatment to detect potential serum electrolyte disturbances, especially hypokalemia (mild hypokalemia was seen in 4/13 patients in the previously performed short-term study). In addition to blood sampling, 24-h urine samples will be collected at the screening, baseline, W12, W48, W96, and EoT and EoS visits. A list of biochemical measurements for blood and 24-h urine samples is included in Table 2. At baseline and at the W12, blood and urine samples will be collected for biobanking. These samples will be shipped to the coordinating center at the end of the trial for additional assays, such as copeptin and MCP-1 measurements. In addition, blood samples will be stored for potential DNA analysis. Biomarker analyses will be performed at the coordinating center after completion of the study to minimize interlaboratory and inter-assay variation. Permission to store samples for biobanking and to perform DNA analysis is optional in this study and will be given separately by patients on an opt-in basis.

#### Vital parameters

Blood pressure and heart rate will be measured at every study visit. Patients will be seated for at least 5 min before measuring blood pressure and pulse rate three times with an automated device. Blood pressure measurements can be performed at home or at the study center as specified in Table 2. Body weight will be measured at every visit, while height will be documented at the screening visit.

#### Quality of life-related questionnaires

Several questionnaires will be completed at baseline, W12, W48, W96, and EoT and EoS visits. The questionnaires used in this study include the Tolvaptan Impact on Polyuria Scale (TIPS), ADPKD Urinary Impact Scale (ADPKD-UIS), EuroQol-5D-5L (EQ-5D-5L), and Short-Form 12 (SF-12). The TIPS (see Supplement 1) is a patient-reported outcome measure designed specifically for this study with input of patient focus groups, the Dutch ADPKD patient organization, and clinical experts. This questionnaire measures the effect of tolvaptan-induced polyuria both on physical and mental health and on social functioning (including participation in daily activities). The other questionnaires have previously been validated [21–23].

#### Data collection

A web-based electronic case report form has been designed for entering study data, to guarantee correct and timely data collection in a central database.

### Intervention

Study treatment consists of daily oral HCT 25 mg (administered as two tablets of 12.5 mg) or matching placebo from baseline until week 156 (EoT). This amount is frequently prescribed in routine clinical practice to treat cardiovascular disorders like hypertension and heart failure, and was demonstrated to be safe and effective in decreasing 24-h urinary volume in the pilot for this study [17]. The study medication (HCT and placebo tablets) is produced by a pharmaceutical company located in the Netherlands. Patients are advised to take the study medication at the same time each day, preferably with the morning dose of tolvaptan. Study medication is dispensed directly to the patient at hospital visits by the trial pharmacy of participating sites. Medication bottles are returned to the study center, and treatment adherence is quantified by counting the number of tablets that are dispensed and returned. Patients will be re-instructed in case of a treatment adherence ≤ 80% or ≥ 120%.

During the study period, study medication can be down-titrated to 12.5 mg for clinical reasons (e.g., hypokalemia, hyponatremia, hypotension, or ≥ 20% drop in eGFR). In case hypokalemia develops during treatment, potassium supplements can be prescribed at the discretion of the treating physician. Antihypertensive medications can be stopped or reduced if hypotension develops, but any RAAS-intervening medications (ACE inhibitors or ARBs) are preferably not altered. If the study medication is down-titrated, the patient will be monitored 2 weeks later. If the clinical or laboratory abnormalities that led to down-titration are resolved, patients may be up-titrated back to 25 mg, continue at 12.5 mg, or the study treatment may be stopped entirely. If the study treatment is stopped prematurely, an early end of treatment discontinuation (EET) visit will be scheduled, and patients will be asked to remain in the study and continue with the visit schedule.

If tolvaptan is stopped during the study period, the investigational product will also be stopped. The patient will, however, be asked to continue study visits and be included in the intention-to-treat analysis.

#### Study blinding and unblinding procedures

Placebo tablets are morphologically identical to HCT tablets. Trial participants, treating investigators (care providers), pharmacy and biomedical personnel, outcome assessors, and the DMC will be blinded to the study treatment. Emergency unblinding for safety reasons is permitted upon request of the local principal investigator and can only be performed by pharmacy personnel involved in the study. All code breaks are reported to the steering committee. At any interim analysis, if there is evidence for differences between the treatment groups in terms of safety or other differences that the DMC finds relevant, the DMC can also be unblinded. The final unblinding of all trial participants is performed after the final dataset is locked.

### Standard care

Study participants will be asked to decline other interventional trials while participating in this study. Hypertension (defined as a systolic blood pressure ≥ 140 mmHg and/or diastolic blood pressure ≥ 90 mmHg) should be treated to a target upper limit of 130/80 mmHg^3^. The first-choice antihypertensive agents in ADPKD patients are ACE inhibitors or ARBs [3, 24]. The use of additional antihypertensive drugs is at the discretion of the treating physician, but diuretics (other than the study treatment) are not permitted. There should be no changes in antihypertensive treatment for 1 month prior to inclusion. Unless it is deemed clinically necessary, antihypertensive treatment will not be changed during the study. Patients will be reminded to adhere to a dietary sodium chloride intake of ≤ 5 g/day and to avoid a protein intake of ≥ 1.3 g/kg/day, as is recommended for ADPKD patients [25]. The tolvaptan dose can be reduced for clinical reasons (at the discretion of the treating physician), but tolvaptan will not up-titrated during the study period to minimize bias for the primary and secondary endpoints.

### Primary endpoint

The primary endpoint is the rate of kidney function decline (expressed as eGFR slope, in mL/min/1.73 m^2^ per year), as calculated by linear mixed-model analysis, using all available on-treatment creatinine values from week 12 to week 156 (EoT) for HCT- versus placebo-treated patients. Creatinine values obtained before week 12 are not used for this calculation because the hemodynamic effects of HCT may influence GFR estimates. eGFR will be calculated using the 2009 CKD-EPI equation [26]. If kidney replacement therapy is started or death occurs during the study, only GFR estimates before these events will be used for analysis.

### Secondary endpoints

Secondary endpoints are related to kidney function, QoL, tolerability, and safety. They include the following:

1. Kidney function
  - Changes in eGFR between baseline and EoS (off-treatment)
  - Incidence of a 30% decrease in eGFR, development of end-stage kidney disease (defined as the start of renal replacement therapy or eGFR decline < 15 ml/min/1.73 m^2^), or death from renal causes
2. QoL
  - Changes in QoL-related questionnaire scores (TIPS, ADPKD-UIS, EQ-5D-5L, and SF-12)
  - Changes in 24-h urine volume
3. Tolerability
  - Tolvaptan and study medication dose and discontinuation rate
4. Safety
  - Changes in serum electrolytes (potassium, sodium, calcium, and phosphate) and blood pressure
  - Incidence of adverse events and mortality

### Exploratory outcomes

Exploratory outcomes include per-protocol analyses for the primary and secondary endpoints and the effects on serum copeptin levels (as a surrogate for vasopressin production) and urine osmolality and on urinary excretion of biomarkers that are associated with kidney damage (e.g., MCP-1).

### Safety considerations

The HYDRO-PROTECT study will use 25 mg of HCT daily, which can be down-titrated if clinically necessary. We expect this dose to be safe based on clinical experience and our previously described pilot study [17]. Since hypertension is a common problem in ADPKD, we anticipate that, even in combination with tolvaptan, hypotensive side effects related to HCT will not occur frequently during the study. The main potential adverse effects from combining tolvaptan with HCT are serum electrolyte changes, in particular, hypokalemia. For that reason, a protocolized blood sample analysis is performed in all patients 2 weeks after the start of study treatment, in addition to the 12-weekly laboratory assessments. In addition, a preliminary safety analysis will be performed after the first 50 patients have completed the W12 visit for this reason. Potassium can be supplemented if required. Given these safety precautions, the starting dose of 25 mg and down-titrating when clinically necessary, instead of an up-titration schedule, is justified. In addition to the risk of electrolyte imbalances, long-term HCT treatment can induce gout, and some studies have also reported an increased incidence of nonmelanoma skin cancer [27, 28]. For that reason, a medical history of active gouty arthritis despite preventive treatment for gout and any form of skin cancer were added as exclusion criteria. Contraceptive counseling and a pregnancy test are performed in women of childbearing potential before study treatment is initiated. Additional pregnancy tests can be performed at the discretion of the treating physician.

### Sample size calculation

The sample size was calculated using the “Optimal Design Software” for mixed models. Assuming an eGFR loss of − 2.72 mL/min/1.73 m^2^ per year (based on the tolvaptan-treated groups in the TEMPO 3:4 and REPRISE trials [7, 8]), a variability of level 1 residuals of 11.85, and variability of level 1 coefficients of 4.69 (both derived from the TEMPO 3:4 trial [7] and validated in our own patient population), a two-sided *α* of 0.05 and a power of 80%, we calculated that 270 patients need to be included for a 3-year period with four visits per year to show a 30% improvement in the rate of kidney function decline (standardized slope difference of 0.82). We deem this 30% improvement clinically relevant because it translates approximately to a 1-year delay in the start of kidney replacement therapy for every 3 years of tolvaptan treatment. To adjust for a 10% drop-out rate, we aim to enroll 300 patients. This expected drop-out rate is based on the results from the TEMPO 3:4 and REPRISE trials. These demonstrated that drop-out was considerable in the first weeks of tolvaptan treatment. However, in patients on stable treatment, like those to be included in the HYDRO-PROTECT study, the drop-out rate was 4.2% (REPRISE) to 9.4% (TEMPO 3:4) [7, 8].

### Statistical analysis

Statistical analyses will be performed after the study is completed. The primary outcome (differences between the treatment groups in eGFR slope expressed in mL/min/1.73 m^2^ per year, calculated using creatinine values from W12 to W156 or EET) will be assessed using linear mixed model analysis for repeated measures. A random intercept and random slope model will be used with unstructured covariance and patient-level random effects. The model will be adjusted for the previously mentioned randomization stratification factors. Between-group differences in the incidence of worsening kidney function (defined as a 30% decrease in eGFR), end-stage kidney disease (start of renal replacement therapy or eGFR decline < 15 ml/min/1.73 m^2^), or death due to renal causes will be investigated using Cox proportional hazard models. Differences in 24-h urine volume and QoL questionnaire scores between the treatment groups will be assessed by ANCOVA analysis. The main analyses will also be performed in a priori-defined subgroups based on age, sex, eGFR, Mayo imaging class, duration of tolvaptan treatment, tolvaptan treatment dose, and dietary intake of sodium and protein (assessed by 24-h urinary sodium and urea excretion, respectively). The subgroups for continuous parameters are based on the group mean. All statistical tests will be two-tailed, and differences are considered statistically significant at a *p*-value < 0.05. All statistical analyses will be performed on an intention-to-treat basis. In addition, exploratory per-protocol analyses will be performed for the primary and secondary endpoints. In case of more than 10% missing data for a primary or secondary endpoint, missing values will be replaced using multiple imputation under the missing at random assumption. These imputed values will be used to perform a sensitivity analysis for that outcome.

### Interim analysis

A preliminary safety analysis of the incidence of hypokalemia and the effects on urinary volume and QoL (but not renal function) will be performed after the first 50 patients have completed the W12 visit. This exploratory safety analysis will be performed because of the incidence of mild hypokalemia in our pilot study [17]. A formal interim safety analysis will be performed by an independent Data Monitoring Committee (DMC) when the 150th patient has completed the W48 visit. This interim analysis is introduced to limit unnecessary exposure of patients to HCT with tolvaptan in case this combination accelerates the decline of renal function. In addition, the incidence of (serious) adverse events in both treatment groups will be reviewed by the DMC. The DMC may advise the steering committee to stop the trial in case of a statistically significantly larger annualized decline in kidney function in the HCT group (expressed as eGFR slope in mL/min/1.73 m^2^ per year) or in case of an increased incidence of serious adverse events in HCT-treated patients. SAEs will be included in the annual safety report, and suspected unexpected serious adverse reactions (SUSARs) are reported electronically through the Eudravigilance portal, as required by the European Clinical Trials Regulation.

### Study organization

A steering committee is responsible for the design, integrity, and progress of the trial. The steering committee is also tasked with implementing any potential protocol modifications, including those that may be recommended by the DMC. The steering committee comprises staff of the UMC Groningen and lead investigators of all participating study centers. An independent DMC has been appointed to carry out the safety-related tasks described above. The DMC consists of three internist-nephrologists and an epidemiologist. All DMC members declared no competing interests. The DMC charter is available as Supplementary material. An academic contract research organization will monitor the progress of the study and the quality of study data. Additionally, a patient advisory committee consisting of several patients with ADPKD was assembled to provide input to the steering committee from a patient perspective.

**Fig. 3:**
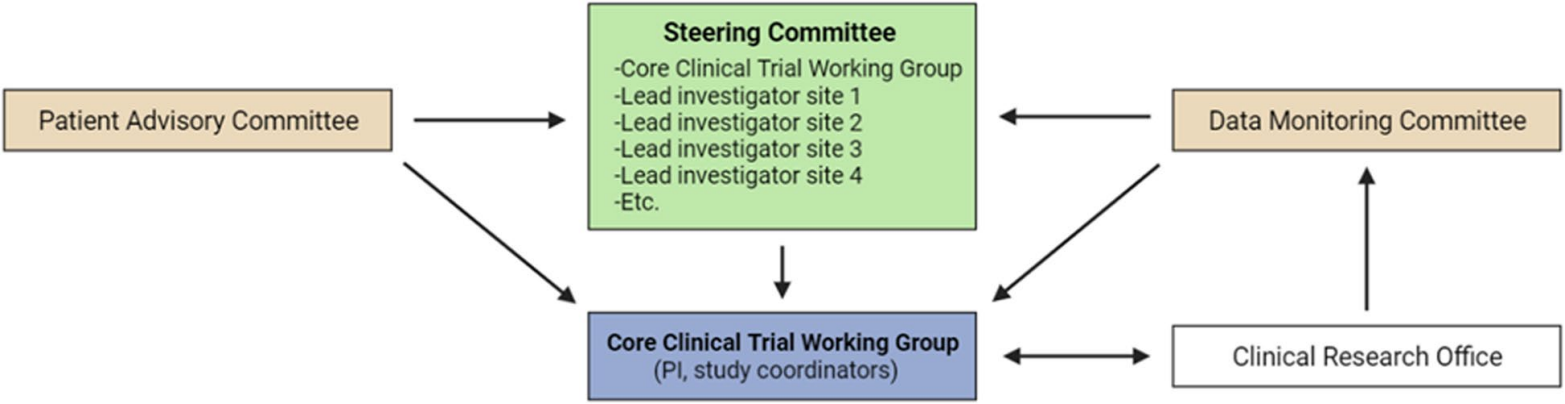
Governance structure. The Core Clinical Trial Working Group (CCTWG) will be responsible for the day-to-day trial management. The steering committee (SC) consists of the CCTWG and the principal investigators of each participating site and is tasked with major decisions regarding the study progress and design. The Data Monitoring Committee (DMC) is responsible for the safety of participants and advises the SC based on the interim analysis. A Patient Advisory Committee has been appointed to advise the SC regarding any patient-related matters. The Clinical Research Office is responsible for, among others, monitoring the trial and for providing safety data that can be assessed by the DMC and be sent to the competent authorities

Figure 3 shows the structure of the study organization and the relationships between its components.

## Discussion

The HYDRO-PROTECT study is the first large-scale randomized clinical trial to investigate the effect of co-treatment with HCT on the efficacy and tolerability of tolvaptan in patients with rapidly progressive ADPKD. If proven effective, the ability to enhance the therapeutic potential of tolvaptan through an additive renoprotective effect and/or increased tolerability would be an important advance, especially since tolvaptan is currently the only available renoprotective treatment for this disease.

Recently, favorable results on 24-h urine volume were reported by an open-label, cross-over pilot trial by Uchiyama et al. that investigated the effect of trichlormethiazide (an alternative thiazide diuretic) in tolvaptan-treated ADPKD patients [29], which corresponds to the results of our pilot study [17]. Although the underlying mechanism behind the anti-aquaretic effect of thiazide diuretics in NDI is not fully elucidated, it is believed that its antagonistic action on the Na^+^-Cl^−^ cotransporter in the distal convoluting tubule and resulting natriuresis leads to a mild state of volume depletion. As a consequence, sodium and water reabsorption in the proximal tubule is increased, so that the total urine volume is ultimately reduced [16]. Another possible mechanism is through the direct upregulation of AQP-2 channels in the collecting tubule in response to HCT [30, 31]. Given the effects of tolvaptan on renal water reabsorption, the main analyses will also be performed in subgroups based on 24-h urine volume, 24-h urine osmolality, duration of tolvaptan treatment, and tolvaptan treatment dose. Separate subgroup analyses will be performed according to baseline eGFR and salt and protein intake, since disease progression is associated with urinary concentrating defects [32, 33] and salt intake [34], and because urinary volume is largely driven by osmolar excretion when renal water reabsorption is impaired [11].

The primary endpoint of this study is the rate of kidney function decline, which is calculated using all available on-treatment serum creatinine values from week 12 to week 156 (EoT) for HCT- versus placebo-treated patients. The slope of eGFR as a measure of kidney function decline is a routine primary outcome in clinical trials in nephrology and is accepted by the EMA and FDA [35]. Week 12 was chosen as a starting point because short-term HCT-induced acute renal hemodynamic changes may influence GFR [36] and thus the primary endpoint. As a secondary endpoint, the difference in renal function will be calculated between baseline and EoS (off-treatment), which will be independent of any hemodynamic effects.

Tolvaptan treatment is exclusively indicated in ADPKD patients with a high likelihood of, or established, rapid disease progression. Although there is no international consensus on the definition of rapidly progressive ADPKD [37], a recently published position statement [38] by the European Renal Association (ERA) defined rapidly progressive disease as an annual eGFR decline of ≥ 3.0 mL/min/1.73 m^2^. In this position statement, an algorithm is presented that estimates the risk of developing rapidly progressive disease based on several disease severity parameters, like kidney function indexed for age, historic eGFR decline, Mayo imaging classification, and PRO-PKD score [38]. Only ADPKD patients who are already on tolvaptan are included in the HYDRO-PROTECT study, which ensures that the trial will be enriched for patients with or at risk for rapid disease progression. This warrants that the renal function decline in our study cohort will be sufficient to detect potential renoprotective effects, even when considering the reduction of eGFR decline as a result of tolvaptan treatment as described in the power analysis.

If HCT co-treatment is indeed associated with a renoprotective effect additive to tolvaptan, it could be hypothesized to result from the antihypertensive properties of HCT. If deemed clinically necessary by the treating physician, changes in antihypertensive medication are allowed. Therefore, we do not expect significant differences in blood pressure to arise between the treatment groups. In addition, the effect of maintaining different blood pressure targets was investigated in early-stage ADPKD (eGFR > 60 mL/min/1.73 m^2^) and later-stage ADPKD (eGFR 25–60 mL/min/1.73 m^2^) by the HALT-PKD A and B studies, respectively [39, 40]. In both, the rates of eGFR decline were similar between the low and standard blood pressure groups. It therefore seems unlikely that any potential renoprotective effects of HCT would be mediated by its antihypertensive actions.

Patients from Belgian centers will need to have an eGFR > 35 mL/min/1.73 m^2^ to participate in the study, because local authorities in Belgium mandate that tolvaptan treatment is stopped when eGFR reaches 25 mL/min/1.73 m^2^. Since cessation of tolvaptan is a secondary endpoint of the trial, the use of an inclusion criterium of eGFR > 25 mL/min/1.73 m^2^ would result in excessive termination of study medication and tolvaptan in these patients and introduce a bias for this endpoint. Accordingly, randomization will be stratified for participating centers and statistical models will be adjusted for clustering.

An interim safety analysis will be performed when the 150th patient has completed the W48 visit. We will not perform an interim analysis for futility since, even in the absence of an effect on eGFR slope, a reduction in 24-h urine volume or increase in QoL could still be a relevant improvement of V2RA therapy.

The HYDRO-PROTECT study has both strengths and limitations. Limitations include the lack of international consensus on either the definition of (risk of) rapidly progressive ADPKD and or the indication(s) for tolvaptan treatment, which could induce patient heterogeneity. In addition, imaging studies are not planned during the trial, since patients are already known to be at high risk of rapidly progressive disease based on their use of tolvaptan. This is because if HCT co-treatment results in a positive effect on total kidney volume (TKV) without any beneficial effect on renal function decline, the clinical use of HCT in addition to tolvaptan would not be justified. Similarly, some previously investigated drugs, for example, mTOR inhibitors and somatostatin analogs, are not widely approved for renoprotection in ADPKD despite interventional trials reporting varying positive effects on TKV [41]. Because of the pragmatic nature of the trial, GFR will be estimated using the creatinine-based CKD-EPI formula instead of using GFR measurements. Although measured GFR remains the gold standard for quantifying renal function, the use of GFR estimates better reflects the real-life situation if HCT is to be implemented for this indication. Strengths of the study include that the effects of HCT are investigated in a real-life setting since participants already receive tolvaptan and that the primary outcome measure is renal function decline, which is clinically more relevant than volumetric outcome measures. Furthermore, the TIPS questionnaire was designed with input from patient focus groups and will therefore measure outcomes that are relevant from a patient perspective. Lastly, the studied intervention (HCT) is inexpensive and readily available, allowing participants to continue or initiate HCT co-treatment after the trial in case of beneficial effects.

In conclusion, the HYDRO-PROTECT study is the first large-scale randomized clinical study to evaluate the effect of co-treatment with HCT on the efficacy and tolerability of tolvaptan in patients with ADPKD.

## Trial status

This article is based on protocol version 9.4 dated August 2023. Recruitment is expected to start in May 2024 and will probably continue until May 2026.

### Supplementary Information

**Additional file 1.**

**Additional file 2.**

**Additional file 3.**

## Data Availability

The dataset obtained during the current study is available to the members of the steering committee and will be available from the corresponding author upon reasonable request. The results of this trial will be published. Once available, the results of this trial will be available from ClinicalTrials.gov under NCT05373264 and will be submitted to the Clinical Trials Information System (CTIS). A summary of the trial results for laypersons will also be submitted in CTIS. The Universal Trial Number for this study is U1111-1283-3529. The SPIRIT 2013 checklist is provided as Supplementary material. Data storage and confidentiality: All participant information and the subject code list are securely stored at the participating study sites in locked file cabinets with limited access. Only pseudonymized data is entered in the electronic case report form (eCRF) within the secure web application “REDCap.” Investigators of the specific participating centers are given access only to their own study participants in this web application. The eCRF includes range checks for data values to promote data quality. Biobanked samples will be shipped to the UMC Groningen at the end of the study in pseudonymized form.
