# Supplementary material for: HYDROchlorothiazide versus placebo to PROTECT polycystic kidney disease patients and improve their quality of life: study protocol and rationale for the HYDRO-PROTECT randomized controlled trial
Source: Trials. 2024 Feb 14;25:120. doi: 10.1186/s13063-024-07952-x (PMC10865620; doi:10.1186/s13063-024-07952-x)
Supplement: Supplementary file 1 — Additional file 1. [file 13063_2024_7952_MOESM1_ESM.pdf]

The HYDROPROTECT study

Data Monitoring Committee (DMC) Charter

TABLE OF CONTENTS PAGE

TITLE PAGE…………………………………………………………………………………………………….. 1

TABLE OF CONTENTS …………………………………………………………………………………… 2

1. INTRODUCTION ....….. 3

2. ROLES AND RESPONSIBILITIES 3

3. BEFORE OR EARLY IN THE TRIAL ....…..... 3

4. COMPOSITION.. 4

5. RELATIONSHIPS……………………………………………………………………………........ 4

6. ORGANISATION OF DMC MEETINGS... 4

7. TRIAL DOCUMENTATION AND PROCEDURES TO ENSURE
CONDIDENTIALITY AND PROPER COMMUNUCATION.. 5

8. DECISION MAKING……………………………………………………………………......... 6

9. REPORTING……………………………………………………………………………………......... 7

10. AFTER THE TRIAL…………………………………………………………………………......... 7

Figure 1: SUMMARY OF HYDRO-PROTECT PROTOCOL.. 8

Appendix 1: POTENTIAL COMPETING INTERESTS FORM.................................... 9

Appendix 2: DMC MEETING REPORT HYDRO-PROTECT...................................... 10

1. INTRODUCTION

The HYDRO-PROTECT trial is an investigator initiated, multicenter, multinational, double-blinded, randomized, controlled clinical trial. The objective of the HYDRO PROTECT trial is to prospectively test whether co-treatment with hydrochlorothiazide can improve the efficacy (slowing kidney function decline) and tolerability (quality of life) of tolvaptan in patients with autosomal dominant polycystic kidney disease (ADPKD) that receive tolvaptan as part of routine clinical care. Patients will be randomized into two groups: treatment with oral hydrochlorothiazide 25 mg once daily or with matching placebo once daily for a total duration of 3 years. The primary endpoint is the rate of kidney function decline (assessed as eGFR slope, in mL/min/1.73m2 per year) as calculated with linear mixed models, using all available creatinine values from month 3 until 36.

The purpose of this document is to describe the roles and responsibilities of the independent Data Monitoring Committee (DMC) for the HYDRO PROTECT study (EUDRACT 2021-005612-61), including the timing of meetings, methods of providing information to and from the DMC, frequency and format of meetings, statistical issues and relationships with other committees.

2. ROLES AND RESPONSIBILITIES

The purpose of the DMC is to safeguard the interests of trial participants, assess the safety and efficacy of the interventions during the trial, and monitor the overall conduct of the clinical trial.

The DMC should inform the Chair of the steering committee if, in their view the results are likely to convince a broad range of clinicians, including those supporting the trial and the general clinical community, that one trial arm is clearly contraindicated,

The purpose of the DMC is to review the trial's progress including updated figures on recruitment, data quality, and main outcomes and safety data. Specific roles include the following:

- meet periodically: before start of the trial, at least yearly and at specific time points: after data are available from 30 patients who have experienced at least one SAE or 6 months after start of the trial (whichever comes first), after the 150th patient has reached 1 year of follow-up, after inclusion of 300 patients and at request of the study coordinators in case of SAEs or high drop-out rates.
- review, give input to and approve the DMC charter
- monitor recruitment figures and losses to follow-up
- monitor compliance with the protocol by participants and investigators
- monitor evidence for treatment harm (e.g. toxicity data, SAEs, deaths)
- decide whether to recommend that the trial continues to recruit participants or whether recruitment should be terminated either for everyone or for some participant subgroups
- suggest additional data analyses
- advise on protocol modifications suggested by investigators or sponsors (e.g. to inclusion criteria, trial endpoints, or sample size)
- monitor planned sample size assumptions
- monitor compliance with previous DMC recommendations
- assess the impact and relevance of external evidence

3. BEFORE OR EARLY IN THE TRIAL

All potential DMC members should have sight of the protocol/outline before agreeing to join the committee. Before recruitment begins the trial will have undergone review by the applicable research ethics committees. Therefore, if a potential DMC member has major reservations about the trial (e.g. the protocol or the logistics) they should report these to the trial office and may decide not

to accept the invitation to join. DMC members should be independent and constructively critical of the ongoing trial, but also supportive of aims and methods of the trial.

The DMC will be given the opportunity to meet before the trial to discuss the protocol, the trial, the analysis plans, future meetings, and to have the opportunity to clarify any aspects with the project leader. The members of the DMC have no contract. However, the members should formally confirm (1) that they agree to be on the DMC and (2) that they agree with the contents of this Charter.

4. COMPOSITION

The members of the DMC for this trial are:

- Prof. dr. Marc Hemmelder (Chair), internist-nephrologist
- Prof. dr. Tom Nijenhuis, internist-nephrologist
- Dr. Ronald van Etten, internist-nephrologist
- Dr. H. Groen, epidemiologist

As characteristic qualifications, DMC members will:

- Work professionally and meet qualifications for their respective professions
- Comply with accepted practices of their respective professions
- Act independent from the SC, IRB/EC, regulatory agencies, principal investigator, co-principal or sub-principal investigator, site investigator, steering committee membership, CEC membership, clinical care of the study subjects, or any other capacity related to trial operations.

The Chair is expected to facilitate and summarize discussions.

The trial statistician, Priya Vart, will distribute the interim report to the DMC and will participate in DMC meetings, guiding the DMC through the report and participate in DMC discussions.

The project leader and/or coordinating investigator may be asked, and should be available, to attend open sessions of the DMC meeting. Other members of the trial management (e.g. Data management) will not usually be expected to attend but can attend open sessions when necessary.

5. RELATIONSHIPS

The role of the DMC is advisory and recommendations made by the DMC are directed at the steering committee.

There will be no payment for DMC members. Members should be reimbursed for travel and accommodation.

DMC members should disclose any competing interests, both financial and non-financial (e.g. involvement in other trials or intellectual investment. Although members may well be able to act objectively despite such connections, complete disclosure enhances credibility. Therefore, a short conflict of interest form should be completed and returned by the DMC members (Appendix 1).

6. ORGANISATION OF DMC MEETINGS

The expected frequency of DMC meeting will be at least once every year.

An initial meeting of the DMC will be proposed in order for the members to review the charter, to form an understanding of the protocol and definitions being used, to establish a meeting schedule, and to review study modification and/or termination guidelines.

Subsequent interim and final review meetings will be held to review and discuss interim and final study data according to the schedule as described below:
-A preliminary safety analysis of the incidence of hypokalemia and the effects on urinary volume and QoL (but not renal function) will be performed after the first 50 patients have completed the W12 visit
- A second safety analysis will occur after data are available from 30 patients who have experienced at least one SAE or 6 months after start of the trial (whichever comes first)
- when the 150th patient has completed 1 year of follow-up (formal interim analysis). This analysis is compares kidney function decline rates between the placebo and treatment groups.
- approximately every 12 months throughout the remainder of the trial upon decision by the DMC

Meetings are face-to-face or by teleconference depending on availability of members and feasibility (also depending on COVID-19 regulations).
The meetings will include both open and closed sessions.

Open session
The open session may be attended by representatives of the sponsor and study investigators. Minutes of the open session will be recorded by the study coordinator. Minutes will be and maintained by the study coordinator in accordance with applicable statutory regulation.

Closed session
The closed session will be restricted to the DMC members. Data which may compromise the integrity of the study (e.g., comparative data) will be analyzed and discussed only in the closed session. Following each meeting, a report of the open and closed sessions will be sent to the sponsor describing the DMC recommendations and rationale for such.

7. TRIAL DOCUMENTATION AND PROCEDURES TO ENSURE CONFIDENTIALITY AND PROPER COMMUNICATION

All DMC members will treat all information relating to data, reports, meeting discussions and minutes as confidential. DMC members do not have the right to share confidential information with anyone outside the DMC, including the PI's.

When there is an open session, open session minutes will be made available to the attendees of the open session during the trial. Accumulating information relating to recruitment and data quality (e.g. data return rates, treatment compliance) will be presented. Toxicity details based on pooled data will be presented and total numbers of events for the primary outcome measure and other outcome measures may be presented, at the discretion of the DMC.

Summary notes (including data reviewed and minutes of discussions) for the closed session will be prepared for each DMC meeting and distributed to the DMC members in a timely manner after each meeting. They will be reviewed and approved at the subsequent meeting. In addition to all the material available in the open session, the closed session material will include safety data by treatment group. As the summary notes for the closed session will contain data by treatment groups (even with treatments designated by code), these will not be forwarded routinely to the steering committee. The statistician will hold these in confidence until the end of the study and after the

treatment code is unblinded, when copies of the summary notes are forwarded to the steering committee. The DMC members should destroy their reports after each meetings. Fresh copies of previous reports will be circulated with the newest report before each meeting when requested so by the DMC.

The DMC reports its recommendations in writing to the Trial Steering Committee (See Appendix 2). This should be copied to the trial statistician and sent via the trials office within two weeks after the DMC meeting.

8. DECISION MAKING

Possible recommendations from the DMC could include:

- No action needed, trial continues as planned
- No action needed, but advance the next review by the DMC
- No action needed, but additional data/review/analyses requested
- To continue the trial and amend the protocol
- Early stopping due to clear harm of a treatment or external evidence
- Stopping recruitment within a subgroup
- Extending follow-up
- Sanctioning and/or proposing protocol changes

A formal interim analysis of safety will be performed when the 150th patient has completed 1 year of the trial, as specified in the protocol. The interim analysis is introduced to safeguard that ADPKD patients will not be exposed to combination of hydrochlorothiazide on top of tolvaptan when this appears to be nephrotoxic. Therefore, the Data Safety and Monitoring Board (DMC) will advise the steering committee to stop the trial in case of:
A larger decline in kidney function in the hydrochlorothiazide group ($p<0.05$).

Every effort should be made for the DMC to reach a unanimous decision. If the DMC cannot achieve this, a vote may be taken, although details of the vote should not be routinely included in the report to the steering committee as these may inappropriately convey information about the state of the trial data. It is important that the implications (e.g. ethical, statistical, practical, financial) for the trial be considered before any recommendation is made. A simple majority of members passes a proposal, motion, or recommendation to the steering committee. If a vote on an issue is tied (e.g., due to a member being unavailable or abstaining), the decision will be made by the DMC Chair.

Effort should be made for all members to attend. Members who cannot attend in person should be encouraged to attend by teleconference. If, at short notice, any DMC members cannot attend at all then the DMC may still meet if at least one member, including the Chair (unless otherwise agreed), will be present. If the DMC is considering recommending major action after such a meeting the DMC Chair should talk with the absent members as soon after the meeting as possible to check they agree. If they do not, a further teleconference should be arranged with the full DMC.

If a DMC member does not attend a meeting, it should be ensured that the member is available for the next meeting. If a member does not attend a second meeting, they should be asked if they wish to remain part of the DMC. If a member does not attend a third meeting, they should be replaced.

Should the DMC recommend early stopping or a major modification of the protocol, the following steps will be undertaken:

- A meeting between the TSC and the DMC to discuss the issues and the preliminary recommendations will be called.
- Following this meeting, the DMC will make a final recommendation.
- The TSC will accept or reject DMC recommendations and report its discussion, as well as its rationale, to the Sponsor and the responsible research ethics committees.
- The TSC has the ultimate responsibility to either stop or modify the trial and to communicate recommendations of the DMC to the (principle) investigators at all study locations and the responsible research ethics committees.

9. REPORTING

The DMC will report their recommendation to the PI of the UMCG by letter, preferably within 3 weeks of the decision. Notes of the DMC meeting will be made by one of the non-chair DMC members.

These findings and recommendations can result from both the open and closed sessions of the DMC. If these findings include serious and potentially consequential recommendations that require immediate action, the chairperson will also promptly notify the Principal Investigator by phone.

A copy from the report will be logged in the Trial Master File.

If the DMC has serious problems or concerns with the SC decision a meeting of these groups should be held. The meeting should be chaired by a senior member of the trials office staff or an external expert who is not directly involved with the trial.

The chair of the steering committee and the central study coordinator will review and respond to the DMC recommendations. The recommendations of the DMC will not be legally binding but require professional consideration by the recipients. If the DMC recommends continuation of the study without modification, no formal response will be required.

However, if the recommendation requests action, such as a recommendation for termination of the study or modification of the protocol, the DMC will request that the principal investigator provides a formal written response stating whether the recommendations will be followed or not and the plan for addressing the issues.

The recommendations of the DMC are not binding. Even in case of recommendations that request action, the SC will make the final decision.

It is recognized that the principal investigator may need to consult with regulatory agencies or other consultants before finalizing the response to the DMC. Upon receipt, the DMC will consider the principal investigator response, resulting in a final decision. Appropriate caution will be necessary during this process to avoid compromising study integrity or the ability of the steering committee to manage the study, should the study continue.

10. AFTER THE TRIAL

Trial results will be published in a correct and timely manner. On request, the DMC will be given the opportunity to read and comment on any publications before submission.

A brief summary of the timings and conclusions of DMC meetings may be included in the body of this paper.

Figure 1: summarizing the HYDRO PROTECT study

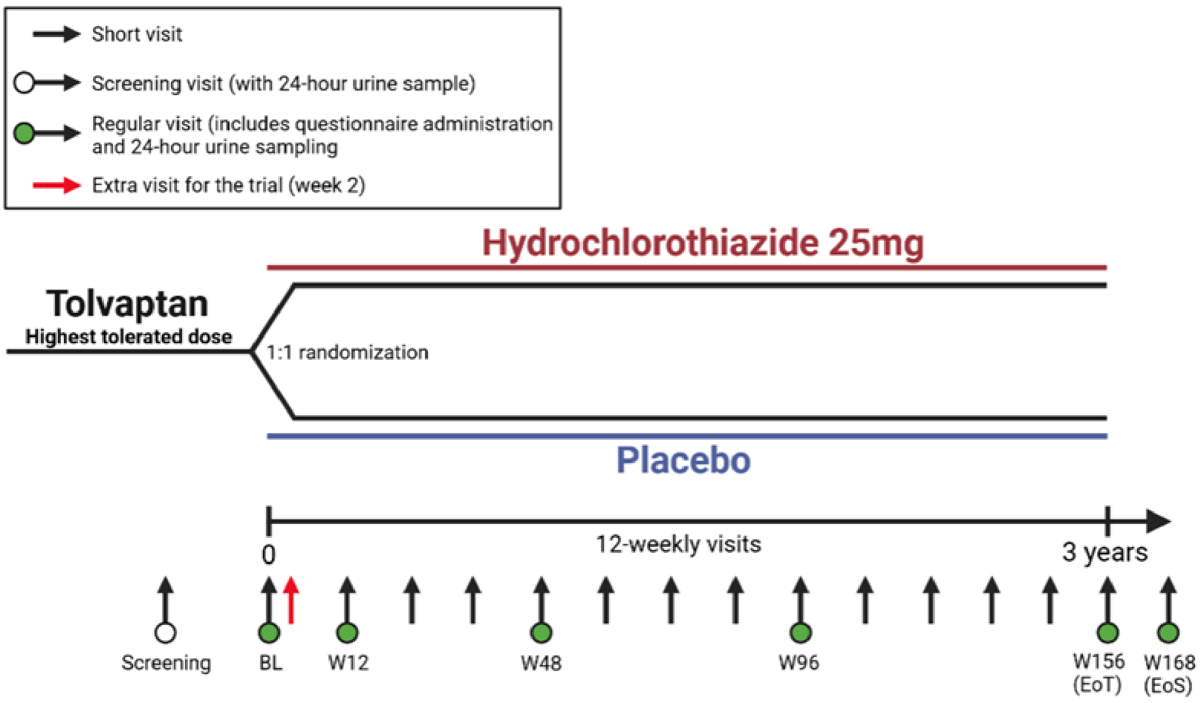

Appendix 1: Potential competing interests of Data Monitoring Committee members for the HYDRO-PROTECT study

The avoidance of any perception that members of a DMC may be biased in some fashion is important for the credibility of the decisions made by the DMC and for the integrity of the trial.

Possible competing interest should be disclosed. In many cases simple disclosure up front should be sufficient. Otherwise, the (potential) DMC member should remove the conflict or stop participating in the DMC. Table 1 lists potential competing interests.

Table 1: Potential competing interests

- Stock ownership in any commercial companies involved
- Stock transaction in any commercial company involved (if previously holding stock)
- Consulting arrangements with the sponsor
- Frequent speaking engagements on behalf of the intervention
- Career tied up in a product or technique assessed by trial
- Hands-on participation in the trial
- Involvement in the running of the trial
- Emotional involvement in the trial
- Intellectual conflict eg strong prior belief in the trial's experimental arm
- Involvement in regulatory issues relevant to the trial procedures
- Investment (financial or intellectual) in competing products
- Involvement in the publication

Please complete the following section and return to the trials office.

☐ No, I have no competing interests to declare

☐ Yes, I have competing interests to declare (please detail below)

Please provide details of any competing interests: ______________________________

____

____

Name: ____________________________

Signed: ___________________________ Date: _______________

Appendix 2: Suggested report from DMC to TSC where no recommendations are being made

[Insert date]

To: Chair of Trial Steering Committee

Dear *[Chair of Trial Steering Committee]*

The Data Monitoring Committee (DMC) for the *[insert trial name]* trial met on *[meeting date]* to review its progress and interim accumulating data. *[List members] attended* the meeting and reviewed the report.

We congratulate the trial organisers and collaborators on the progress and conduct of the trial and the presentation of the data. The trial question remains important and, on the basis of the data reviewed at this stage, we recommend continuation of the trial according to the current version of the protocol *[specify protocol version number and date]* with no changes.

We shall next review the progress and data *[provide approximate timing]*

Yours sincerely,

*[*Name of meeting Chair*]*

Chair of Data Monitoring Committee

On behalf of the DMC (all members listed below)

DMC members:

(1) [*Insert name and role*]

(2) [*Insert name and role*]

(3) [*Insert name and role*]
