# Supplementary material for: HYDROchlorothiazide versus placebo to PROTECT polycystic kidney disease patients and improve their quality of life: study protocol and rationale for the HYDRO-PROTECT randomized controlled trial
Source: Trials. 2024 Feb 14;25:120. doi: 10.1186/s13063-024-07952-x (PMC10865620; doi:10.1186/s13063-024-07952-x)
Supplement: Supplementary file 2 — Additional file 2. [file 13063_2024_7952_MOESM2_ESM.pdf]

Subject Information for Participation in Medical Scientific Research

HYDRO-PROTECT study (EU CT: 2022-500210-26-00)

Hydrochlorothiazide in Combination with Tolvaptan for Preserving Kidney Function and Quality of Life in Autosomal Dominant Polycystic Kidney Disease (ADPKD)

Dear Sir/Madam,

We kindly invite you to participate in a medical scientific research study.
Participation is voluntary, but we do require your written consent to participate. Before you decide whether you want to take part in this study, you will receive an explanation of what the research entails. Please read this information carefully and ask the researcher for clarification if you have any questions. Please feel free to discuss study participation with your partner, friends, or family as well.

1. General Information

This research is conducted in multiple University Teaching hospitals in the United Kingdom and the European Union. In the United Kingdom, this study is conducted in collaboration with the University of Cambridge and the University of Sheffield. A total of 300 subjects are required for this research. The study was approved by a Medical Ethics Review Committee.

2. Goal of the study

Previous research indicates that adding the medication hydrochlorothiazide (HCT) to tolvaptan (brand name: Jinarc) reduced urine production and thereby improved the quality of life for patients with polycystic kidneys. Additionally, we found evidence suggesting that adding HCT to tolvaptan slows down the decline in kidney function. In this current project, we aim to investigate these beneficial effects of adding HCT to tolvaptan in a study involving 300 patients over a three-year period.

3. Background of the study

You are currently using tolvaptan (Jinarc) as a treatment for autosomal dominant polycystic kidney disease (ADPKD). The goal of tolvaptan treatment is to delay kidney failure. The most significant side effect of tolvaptan is frequent urination, with people often urinating up to 6 liters per day. This can have a significant impact on your daily life and is the primary reason some people discontinue tolvaptan treatment. Tolvaptan is currently the only treatment that has been shown to slow cyst growth and delay kidney function decline. Therefore, we are looking for ways to improve the treatment. We suspect that adding HCT to the treatment may reduce frequent urination, thereby improving sleep quality and overall quality of life. Moreover, there is evidence that this combination may more effectively slow down the decline

in kidney function. Please refer to Attachment D for an explanation of why we anticipate that hydrochlorothiazide may have favorable effects when used in conjunction with tolvaptan.

4. Participation in the study

If you decide to participate in the study, it will last for approximately 3 years in total. Participation can only begin after you have been deemed suitable for participation, and the attached Consent Form (Attachment E) has been completed and received by us.

Suitability Assessment

After you have provided written consent to participate in the study, we will assess whether you are eligible. This is called 'screening.' The researcher will conduct a physical examination, measure your weight, height, blood pressure, and heart rate, and perform blood tests. The researcher will also inquire about your medical history. Reasons you may not be able to participate include abnormal blood values or other conditions that pose a risk when using hydrochlorothiazide.

Sometimes, during the suitability assessment, we may find something that requires further medical investigation. We will always inform you of such findings. Further investigation will be carried out by your own general practitioner or specialist, and the costs will be covered by your own insurance.

Treatment

In addition to the standard treatment with tolvaptan that you are already receiving as part of your regular treatment, we will treat you with hydrochlorothiazide for three years, or with an inert substance (placebo) for three years. The placebo tablets look identical to the real hydrochlorothiazide tablets from the outside. Neither you nor the researcher will know whether you are receiving hydrochlorothiazide or the placebo. However, if it is essential for your health, this information can be looked up. The treatment consists of two pills to be taken once daily in the morning. A random draw will determine whether you are treated with hydrochlorothiazide or placebo. The chance of being assigned to the placebo group is 50% (1 in 2; like flipping a coin). This random draw takes place a few weeks after the suitability assessment during a visit to the hospital (the 'baseline' visit). You will receive the medication during this visit and immediately begin the investigational treatment.

We are comparing the effectiveness of hydrochlorothiazide with the placebo. The use of a placebo group is necessary in this study to effectively assess the effectiveness of hydrochlorothiazide. We understand that 3 years is a lengthy period. The study's duration is necessary because it takes time to obtain a clear picture of the rate of kidney function decline.

Visits and measurements

During the research period, you will visit the hospital every 3 months, plus one additional visit, for a total of 17 visits. With the exception of the visit 2 weeks after starting treatment (which

can also be a phone appointment with blood tests conducted locally), all other visits can be combined with your standard nephrologist appointments. Blood will be drawn at each appointment, typically the same amount as during your regular check-up. Additionally, you will complete a few questionnaires at some visits. Over the course of 3 years, you will need to collect 24-hour urine samples seven times. You can find a detailed schedule of the study and an overview of measurements per visit in Attachment C.

If you have a blood pressure monitor at home, you may not need to come to the hospital for some visits. Please discuss this with the researcher.

Differences from standard care

During the study, only one extra visit is required in addition to your regular check-ups with your nephrologist. You will also fill out four questionnaires a few times, and extra blood samples will be taken at some visits. The questions concern your health perception and urination frequency, totaling 55 questions. Filling out these questionnaires will take you approximately 20 minutes each time. These questionnaires will be sent to your email address, and it is expected that you complete them before some of the hospital visits.

Collection and storage of blood and urine samples (biobank)

We would like to collect some additional blood and urine samples on two occasions during the study for storage. This will involve a total of 6 blood tubes and 2 tubes of 24-hour urine. We want to store these samples to potentially answer future questions, such as research on substances that affect the course of the disease (biomarkers). We would also like to be able to conduct genetic research (DNA diagnostics) on these samples. Genetic diagnostics will only be directly related to the research questions of this study, and may involve genes related to polycystic kidney disease or genes that impact the effectiveness of medications for the disease. At the end of the study, these samples are transferred and stored in the Central Freezer Facility at the University Medical Center Groningen, the Netherlands.

You have the option to provide separate consent on the consent form (Attachment E) for the storage of these samples, their use in future research, and genetic research. Providing this consent is not mandatory for participation in this study.

5. What is expected from you during study participation?

We would like to establish the following agreements with you to ensure the smooth progress of the study:

We will ask that you:

- Take the investigational medication as instructed.
- Do not participate in any other medical scientific research that investigates medications or tries to reduce the side effects of tolvaptan. This could affect the results.
- Attend visits as scheduled.

- Carry the study participant card with you. This card states your participation in this study and provides information on who should be contacted in case of an emergency. Please present this card when visiting other healthcare providers.
- Make an effort to adhere to the recommended maximum salt intake of <5 grams per day. This is the standard advice for patients with ADPKD.
- It is possible that you may want to discontinue tolvaptan and/or HCT during the study. Naturally, this is allowed. In that case, we will ask whether we can continue collecting data during your regular visits to the nephrologist.

It is important that you contact the researcher in the following situations:

- Before starting any other medications, including homeopathic remedies, herbal remedies, vitamins, and/or over-the-counter medications.
- If you are admitted to or treated in a hospital.
- If you experience sudden health issues.
- If you no longer wish to participate in the study.
- In case of changes to your phone number, address, or email address.

Pregnancy

You are being treated with tolvaptan as part of standard care for polycystic kidneys. This medication should not be used during pregnancy, and this restriction also applies during the study period. The use of hydrochlorothiazide during pregnancy can also pose a risk to the unborn baby. Pregnant or breastfeeding women cannot participate in this study. Women should also not become pregnant during the study period. These restrictions are similar to those of standard tolvaptan treatment. There are no additional limitations. Women who could become pregnant (‘women of childbearing potential’) must undergo a pregnancy test before starting the study treatment.

The following methods are acceptable forms of highly effective contraception:

- Oral contraception
- Injectable, implantable, or intravaginal methods or hormone therapy with patches
- Hormonal intrauterine or intrauterine systems (hormonal IUDs)
- Bilateral tubal occlusion (closure of both fallopian tubes)
- Having a sterilized or infertile partner, if your partner is your only sexual partner and sterilization has been confirmed as successful.
- If you are in menopause or permanently infertile due to surgery (e.g., if your ovaries or uterus have been surgically removed), you do not need to use contraception.

It is not necessary to use any of the above-mentioned contraception methods if you do not engage in heterosexual intercourse or do not plan to have sexual contact during the study period. If you have any questions about contraception, please contact your study doctor or study nurse.

Unforeseen pregnancy

If you become pregnant during the study, please inform the researcher immediately. Your study participation will end and additional monitoring may be performed during the pregnancy.

6. Possible side-effects

The investigational medications can cause side effects.
Possible side effects may include:
-Disturbance of electrolyte levels in the blood, particularly sodium and potassium, which can lead to muscle weakness or muscle cramps.
-Low blood pressure.
-Dizziness when standing up.
-Palpitations (heart palpitations).
-Loss of appetite.
-Gastrointestinal issues (such as nausea, vomiting, diarrhea, and abdominal cramps)

Adverse consequences of study-related procedures
Blood draws can be painful and may result in bruising.

7. Possible advantages and disadvantages of study participation

Participating in the study can have both advantages and disadvantages. Below, we list them for your consideration, and it is important to think carefully about them before participating in this study.

A potential advantage of participating in the study and receiving hydrochlorothiazide treatment is that you may experience fewer side effects. If you are randomized to receive a placebo, you will not have a personal benefit. The advantage is that we gain knowledge that could potentially lead to better treatment for ADPKD in the future and help alleviate the frequent urination associated with tolvaptan.

Disadvantages of participating in the study may include:
-Taking extra medication for three years (2 tablets per day).
-Possible side effects of the medication, as described above.
-Approximately 64 ml of extra blood drawn throughout the entire study.

Participation in the study also means:
-Spending extra time, such as filling out questionnaires.
-One extra visit to the hospital or a local laboratory for blood collection.
-(Optional): Three extra blood tubes will be collected for storage during two of the routine blood draws.
-(Additional) collection of 24-hour urine samples.
-Adherence to specific appointments and requirements.

All of these aspects have been described in detail in sections 4, 5, and 6.

8. If you do not want to participate or wish to discontinue your participation

You decide whether or not to participate in the study. Participation is voluntary. If you choose not to participate, you will continue to receive standard treatment for your polycystic kidneys, and your clinic visits will proceed as usual.

If you do decide to participate, you can change your mind and withdraw from the study at any time, even during the course of the research. In such a case, you will resume standard treatment for your polycystic kidneys, and your clinic visits will return to their usual schedule. You are not required to provide a reason for discontinuing your participation, but you must promptly inform the researcher of your decision. The data collected up to that point will be used for the research. If there is new information about the study or the medication used in this study that is important for your health, the researcher will inform you. If you choose to discontinue the study, you will be asked if your future blood values can still be used. This is important for the quality of the research.

9. What happens after your participation has ended?

Your participation in the study will end when:

- All visits according to the schedule are completed
- You decide to discontinue
- You become pregnant
- The researcher determines it's better for your health to stop participating
- The UMCG, a government agency, or the reviewing medical ethics committee decides to terminate the study

The entire study will conclude when all participants have completed it. After processing all the data, the researcher will inform you about the study's key findings. At that time, we can inform you whether HCT was effective in slowing kidney function decline and reducing urine volume. In some cases, it may be challenging to determine if it was effective for you specifically. It may take approximately a year to process and fully analyze all the data. The researcher can also inform you whether you were treated with hydrochlorothiazide or a placebo. If you prefer not to know, you can inform the researcher of that choice.

If you have participated in the study, you may be contacted in the future for additional questions or to participate in new research. You can provide separate consent for this on the consent form.

10. Use and Storage of Your Data and Body Material

For this study, it is necessary to collect and use your medical data to answer the study's research question. Each participant will be assigned a code that will be used on the data. Your name and other personal information that could directly identify you will be omitted. All your data will remain confidential. Only the researcher(s) at the participating hospital will know which code belongs to you.

Your data

If you choose to participate in the study, you also give consent to collect, use, and store your data. The local researcher will store the following data:

-Your gender

-Your ethnicity; this information is necessary for this study because ethnicity is considered when calculating kidney function.

-Your date of birth

-Health-related data

-(Medical) data collected during the study

The researcher will store your data for 25 years, which is a mandatory retention period. Additionally, we would like to use medical data for future research related to the current study, such as research on the disease course of polycystic kidneys. You can provide separate consent for this on the consent form (Attachment E).

If you consent to storing bodily materials (blood and urine) as described on page 3, we will store them at the UMC Groningen. They will be kept for 25 years to allow for new assessments related to this research throughout the study. Once they are no longer needed, your bodily materials will be destroyed.

You can also provide separate consent on the consent form to be approached for potential follow-up studies related to cystic kidneys after the conclusion of this study.

How do we protect your privacy?

To protect your privacy, we assign a code to your data and bodily materials. Only this code is used on all your data and bodily materials. The code's key is securely stored at the hospital. When processing your data and bodily materials, we will only use this code. Even in reports and publications about the study, no one can trace your data back to you.

Who can access your data?

Some individuals may have access to your name and other personal information without the code. These may be data specifically collected for this research or data from your medical record.

These are people who verify that the researchers are conducting the study correctly and reliably. These individuals may access your data:

• Members of the committee overseeing the safety of the research.

• An auditor hired by the researchers.

• National and international regulatory authorities.
These individuals keep your data confidential.

Subsequent use of data and/or bodily materials
We would like to keep your data, including details such as your age, gender, ethnicity, study examination results, and blood and urine samples. This information may be useful for future research. This includes research related to this study, such as additional investigations into disease progression or further research on the side effects of tolvaptan and hydrochlorothiazide in patients with cystic kidneys. Bodily materials will be stored in a coded manner and will not be traceable to you. The data will be retained for 25 years after the last visit of the last participant.

This study is also listed in a registry of medical scientific studies, namely ClinicalTrials.gov. This website does not contain information that can be traced back to you as an individual. However, after the study concludes, the website may display a summary of the results. You can find this study at https://clinicaltrials.gov/ct2/show/NCT05373264. In addition, a summary of the research results, including a layman's summary, will be made available in the public section of the European database.

Using your data for other research
Your collected data and (remaining) bodily materials may also be relevant for other scientific research in the field of cystic kidneys. Therefore, your data and bodily materials will be stored for 25 years at the hospital. On the consent form, you can indicate whether you agree to this. If you do not provide consent, you can still participate in this study, and you will receive the same care.

What happens in case of unexpected health findings?
During the study, we may accidentally discover something that is not directly related to the study, but is relevant to your health or the health of your family members. In such cases, the researcher will contact you and your general practitioner or medical specialist. You will then discuss with your general practitioner or specialist what needs to be done. The costs will be covered by your own health insurance. You give consent on the form for informing your general practitioner or specialist.

Can you withdraw your consent for data usage?
You can withdraw your consent for the use of your data at any time. Please inform the researcher in this case. However, please note that if you withdraw your consent and researchers have already collected data for a study, they may still use this data. Regarding your bodily materials, if you withdraw your consent, the researcher will destroy them. However, if measurements have already been made using your bodily materials, the results may still be used.

11. Trial participant insurance

For everyone participating in this study, insurance has been taken out. The insurance covers damage caused by the study. Contact information of the insurer can be found in Attachment B.

12. Informing your general practicioner, medical specialist and pharmacist

We always send a letter to your general practitioner, treating specialist, and pharmacist to inform them that you are participating in the study. This is for your own safety. If you do not agree with this, you cannot participate in this study. In case of medical emergencies, we may contact your (family) doctor, for example, regarding your medical history or medication use.

13. Financial compensation

The study medication, additional tests, and treatment for the study will not cost you anything. Your regular check-ups, including the study visits, are still covered by your insurance. You will not be paid for participating in this study. Since measurements for the study are conducted during your regular clinic visits, you will not receive a travel reimbursement.

14. Questions

If you have questions, you can contact the researcher. If you wish to file a complaint, please contacts the complains officer at your study hospital. You can find their contact details in Attachment A. The researcher can also give you their contact information.

15. Written consent form

After you have had sufficient time to consider (up to a maximum of 4 weeks), you will be asked to decide on participating in this study. If you agree, we will ask you to confirm this in writing on the accompanying consent form. By providing your written consent, you indicate that you have understood the information and agree to participate in the study. The signed form will be retained by the researcher. You will receive a copy of this information letter and consent form.

Thank you for your time and attention.

16. Attachments to this information letter

A. Contact details

B. Insurance information

C. Visit schedule

D. Background of this research

E. Consent form

Attachment A: Contact details for <study center>

<Name of Study Center>
<address>
<telephone number>

<emergency contact instructions>

Principal Investigator
<Name>
<Telephone number>
<optional: email>

Trial coordinators/study personnel
<Name>
<Telephone number>
<email>

<Name>
<Telephone number>
<email>

Complaints:
If you have a complaint about the conduct of the research, you can report it to the principal investigator. If you do not wish to do so, you can contact the independent complaints officer at: <telephone number> or <email>.

Data Protection Officer
For more information about your rights regarding collection and processing of your data, you can contact the Data Protection Officer at <email>.

Attachment B: Insurance information

The UMCG (University Medical Center Groningen) has taken out insurance for everyone in the United Kingdom participating in the study. The insurance covers the damage you incur due to your participation in the study. This includes damage that occurs during the study or within 4 years after the study. You must report any damage to the insurer within 4 years.

If you have suffered damage due to the study, please report it to this insurer:

The insurer of this study is:<br>**-** CNA Insurance Company (Europe) S.A.<br>-35F, avenue John F. Kennedy, L-1855 Luxembourg<br>-For the UK: Floor 13, 20 Fenchurch Street, London, United Kingdom<br>-Policy number: ML6BBA3439AX<br>-Telephone: +44 20 7743 6800<br>E-mail: melissa.bixby@cnahardy.com

Attachment C: Visit schedule

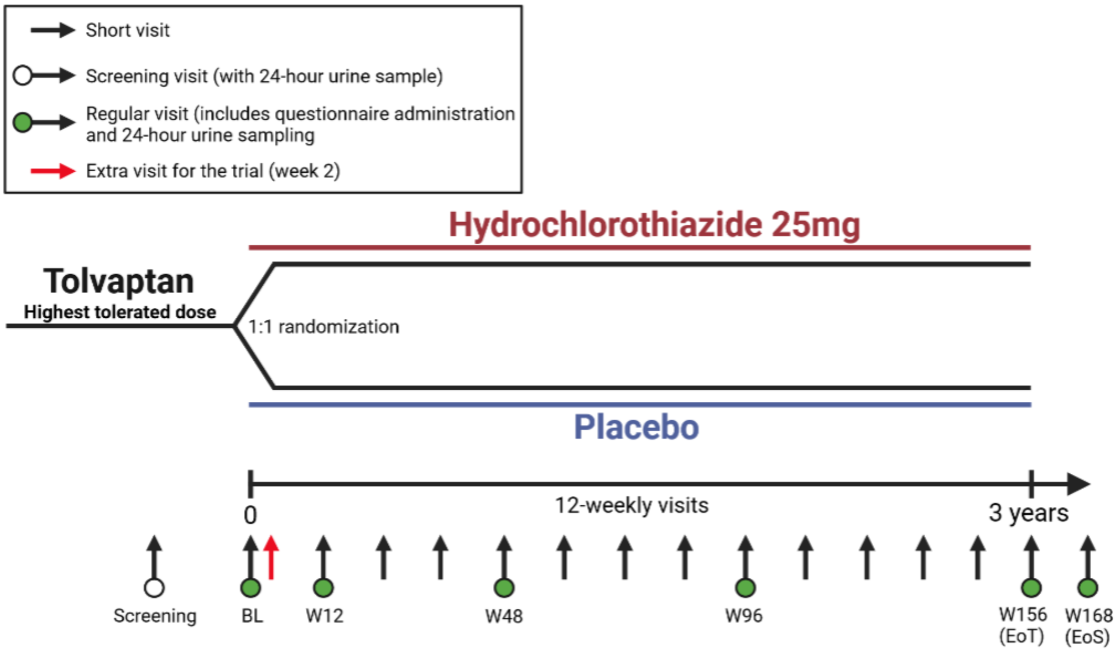

You will be treated with hydrochlorothiazide or a placebo (an inactive substance). Randomization will determine which treatment you receive, and the treatment will last for three years.

Visits

There will be a total of 17 visits, each lasting approximately 15 minutes to half an hour. One visit will occur at the beginning of the study, one after two weeks, and then every three months for three years. Except for the visit that takes place two weeks after starting the treatment, all visits will be combined with your regular check-ups with the nephrologist.

During the visits, the following will take place:
-A physical examination will be conducted, including measuring your weight and blood pressure. During some visits, the study personnel will also perform some standard examinations, such as listening to your heart and lungs and examining your abdomen.
-Blood samples will be taken, just like during your regular visits to your nephrologist.

Extra visit

Two weeks after starting the study medication, there will be an extra visit. In consultation with the researcher, this can also be a blood draw at a location near you with a telephone appointment.

Urine collection

You will be asked to collect 24-hour urine samples a total of seven times. This includes during the eligibility screening, at the start of the study (baseline), after three months,

annually, and after discontinuing the study medication. Collecting 24-hour urine is a standard part of clinical care. You will be provided with urine containers to take home, and you must collect all your urine over a 24-hour period in these containers. You will then bring the containers to the hospital, where they will be collected, weighed, and subjected to laboratory tests. During the same visits, four questionnaires will also be administered (except during the screening visit). Collecting the 24-hour urine during the eligibility screening is intended to determine if it is feasible for you to collect a 24-hour urine sample because it can be a large volume of urine. No analyses will be performed on that sample. If you are unable to collect a 24-hour sample, for example, because the volume is too large to transport, we will explain how you can mix the urine and bring a portion to the study.

Attachment D: Study background

You are using tolvaptan as a treatment for your polycystic kidneys. The most common side effect is increased urination, averaging around 6 liters per day. Because this is bothersome and often the primary reason people discontinue tolvaptan, we aim to reduce this excessive urination. Another goal of the study is to improve the effectiveness of tolvaptan in delaying kidney failure.

Why do we expect hydrochlorothiazide to work?

In a small study conducted at the UMCG, we observed that patients with polycystic kidneys who were treated with tolvaptan experienced a 25% reduction in urination when they were also treated with hydrochlorothiazide. Additionally, certain harmful substances in the blood decreased that may contribute to the loss of kidney function. This is a potential indication for us that adding hydrochlorothiazide may further slow down the decline in kidney function..

Attachment E: Written consent form

The following is a summary of your consents regarding participation in the HYDRO-PROTECT study:

- I have read the information letter. I also had the opportunity to ask questions, and my questions have been adequately answered. I had enough time to decide whether I want to participate.
- I understand that participation is voluntary, and I can decide at any time not to participate in the study or to discontinue my participation.
- I give the researcher permission to inform my general practitioner, treating specialist(s), and pharmacist that I am participating in this study.
- I give the researcher permission to request medical information from my general practitioner and treating specialist(s).
- I give the researcher permission to provide my general practitioner or specialist with information about unexpected findings from the study that are relevant to my health.
- I agree to share my data with other researchers and Research Data Support UMCG staff, both nationally and internationally, even outside the European Union, on the condition that this data is exchanged in a coded (pseudonymized) form. I understand that the data will be retained for 25 years.
- I understand that some individuals listed in this information letter may have access to my data for the purpose of study monitoring. I grant these individuals permission to access my data for this purpose.
- I consent to the use of my email address to send me the questionnaires for the study.
- (For women who could become pregnant): I understand that I must not become pregnant during the study. I agree to undergo a pregnancy test before starting the study treatment.
- I give the researchers permission to inform me after the study about which treatment I received or which group I was in

1. I want to participate in this study and consent to the abovementioned conditions

Yes ☐ No ☐

The following questions are optional for participating in the study. You are not required to answer these questions with a 'yes' in order to participate in the study.

2. I give permission to use the medical data collected during this study for future research. The future research is related to the research questions in this study.

Yes ☐ No ☐

3. I consent to the collection, gathering, and storage of blood and urine samples, as described in this information sheet. The samples will be kept for 25 years.

Yes ☐ No ☐

4. I consent to the use of my stored blood and urine samples (mentioned in point 3) for potential future research related to the research questions in this study.

Yes ☐ No ☐

5. I consent to the performance of genetic research (DNA diagnostics) on my stored blood and urine samples. This diagnostics will only be used to answer relevant research questions for this study.

Yes ☐ No ☐

6. I give permission to, after this study has ended, be asked by the local researcher to participate in future research related to polycystic kidney disease

Yes ☐ No ☐

Your name (study participant): ………………………………..
Signature: ……………………… Date : __ / __ / __

I declare that I have fully informed this participant about the mentioned research.

If information arises during the study that could affect the participant's consent, I will promptly inform them.

Name of researcher (or delegated representative):……………………………….
Signature:………………………   Date: __ / __ / __

The participant will receive a complete information letter along with a signed copy of the informed consent form.
